# Computational design, chemical synthesis, and biological evaluation of a novel ERK inhibitor (BL-EI001) with apoptosis-inducing mechanisms in breast cancer

**DOI:** 10.18632/oncotarget.3105

**Published:** 2015-01-27

**Authors:** Bo Liu, Leilei Fu, Cui Zhang, Lan Zhang, Yonghui Zhang, Liang Ouyang, Gu He, Jian Huang

**Affiliations:** ^1^ State Key Laboratory of Biotherapy/Collaborative Innovation Center of Biotherapy, West China Hospital, Sichuan University, Chengdu 610041, China; ^2^ School of Traditional Chinese Materia Medica, Shenyang Pharmaceutical University, Shenyang 110016, China; ^3^ Collaborative Innovation Center for Biotherapy, Department of Pharmacology & Pharmaceutical Sciences, School of Medicine, Tsinghua University, Beijing 100084, China

**Keywords:** Extracellular signal-regulated kinase 1/2 (ERK1/2), ERK inhibitor (BL-EI001), Apoptosis, Mitochondrial pathway, Breast cancer

## Abstract

Extracellular signal-regulated kinase1/2 (ERK1/2) plays a crucial role in the resistance of apoptosis in carcinogenesis; however, its targeted small-molecule inhibitors still remain to be discovered. Thus, in this study, we computationally and experimentally screened a series of small-molecule inhibitors targeting ERK toward different types of human breast cancer cells. Subsequently, we synthesized some candidate ERK inhibitors, identified a novel ERK inhibitor (BL-EI001) with anti-proliferative activities, and analyzed the BL-EI001/ERK complex. Moreover, we found that BL-EI001 induced breast cancer cell apoptosis via mitochondrial pathway but independent on Ras/Raf/MEK pathway. In addition, we carried out proteomics analyses for exploring some possible BL-EI001-induced apoptotic pathways, and further found that BL-EI001-induced apoptosis affected ERK phosphorylation in breast cancer. Further, we found that BL-EI001 bear anti-tumor activities without remarkable toxicities, and also induced mitochondrial apoptosis by targeting ERK *in vivo*. Taken together, these results demonstrate that *in silico* design and experimental discovery of a synthesized small-molecule ERK inhibitor (BL-EI001) as a potential novel apoptosis-inducing drug in the treatment of breast cancer.

## INTRODUCTION

Mitogen-activated protein kinases (MAPKs) are serine/threonine-specific protein kinases that can regulate cell growth, differentiation, proliferation and apoptosis [1, 2]. The classic MAPK family consists of 4 subfamilies: extracellular signal-regulated kinases (ERKs), the c-Jun-N-terminal kinases (JNKs), the p38MAPKs and ERK-5. Amongst them, ERKs (ERK1/2) are the important signal transduction enzymes, identified by the specificity motif TEY (Thr-Glu-Tyr) sequence in their activation sites [3]. ERKs are terminal kinases in the cellular transduction pathways connecting the cell surface receptors to the nuclear signaling pathway mechanisms [4]. The ERK signaling pathway has been known to play a crucial role in carcinogenesis, including resistance of apoptosis by activating several anti-apoptotic protein (e.g., Bcl-X_L_ and Mcl-1) or inhibiting pro-apoptotic proteins (e.g., Bim, Bad and caspase 9) [5]. Apoptosis is a tightly regulated multi-step pathway that is responsible for cell death not only during development, but in adult multicellular organisms [6]. Deregulation of apoptosis can lead to an accumulation of undead cells and contribute to cancer development [7]. Thus, inhibiting the ERK pathway may be a potential therapeutic strategy for preventing the resistance of apoptosis in cancer cells.

Hitherto, cancer drug development has profited from accumulating understanding of apoptotic mechanisms for several years, and till now, some small-molecule compounds targeting the ERK pathway have been evaluated *in vitro* and *in vivo* tests. Initial small-molecule inhibitor development has been focused on pyrazolopyridazines such as FR180204, which is a modest ERK inhibitor [8]. Afterwards, a pyrimidylpyrrole-based ERK inhibitor VTX-11e, has been reported to be a potent ERK inhibitor with oral bioavailability [9]. The strong activation of ERK in apoptosis resistant tumors suggests direct targeting of ERK as an attractive strategy for clinical cancer trials. At least two ERK inhibitors are in phase I studies, including MK8353, a clinical grade analog of SCH772984, and BVD-523 [10]. Recently, SCH772984 has been shown to be a selective and potent ERK1/2 inhibitor which preferentially affects cell survival of BRCA2-deficient breast cancer cells, as well as also induces apoptosis and cell cycle arrest in BRAF-mutant or non-BRAF-mutant melanoma [10–12]. However, discovery of novel ERK inhibitors and elucidation relevant molecular mechanisms still remain in its infancy for the current cancer therapy.

Thus, in this study, we identified a novel small-molecule ERK inhibitor (BL-EI001), supported by a series of computational design and experimental validation, indicating that BL-EI001 may be a promising apoptosis-inducing drug for future breast cancer therapy.

## RESULTS

### Molecular modeling, docking screening and anti-proliferative activities of ERK inhibitors toward breast cancer cells

**Figure 1:**
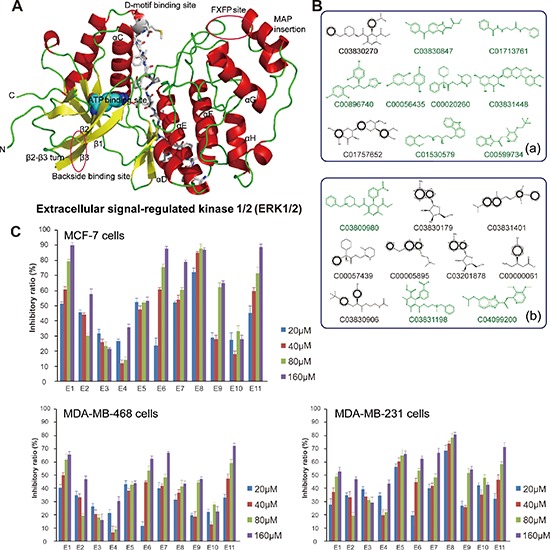
Molecular modeling, docking and anti-proliferative screening of candidate small-molecule compounds targeting ERK1/2 **(A)** The molecular structure of ERK1/2; **(B)** The top ten candidate small molecule compounds targeting ERK1/2 from Drugbank and ZINC, respectively; **(C)** Anti-proliferative activities of candidate compounds (E1-E11) toward MCF-7 cells, MDA-MB468 cells and MDA-MB231 cells, respectively.

In this study, we carried out the molecular modeling of ERK1 based on its crystallographic structure (Figure 1A). We screened the structure-based candidate small-molecule compounds that could target ERK from Drugbank and ZINC, respectively. Subsequently, we achieved some small-molecule compounds from Drugbank that could bind their target ERK1/2 very well (Table S1). In addition, we achieved other small molecule compounds from ZINC that could also bind their target ERK1/2 very well (Table S2). Thus, we show the top eleven compounds from Drugbank and ZINC for further studies (Figure 1B). Next, we obtained eleven compounds through the commercial purchase or chemical synthesis named E1-E11. Then, the MTT assay was conducted with these compounds in MCF-7, MDA-MB468 and MDA-MB231 cells, respectively. As we could see, compound E1 had good inhibitory activity, and the inhibition was dose-dependent (Figure 1C).

### Synthesis of a novel ERK inhibitor (BL-EI001) and its molecular dynamics stimulations with ERK

**Figure 2:**
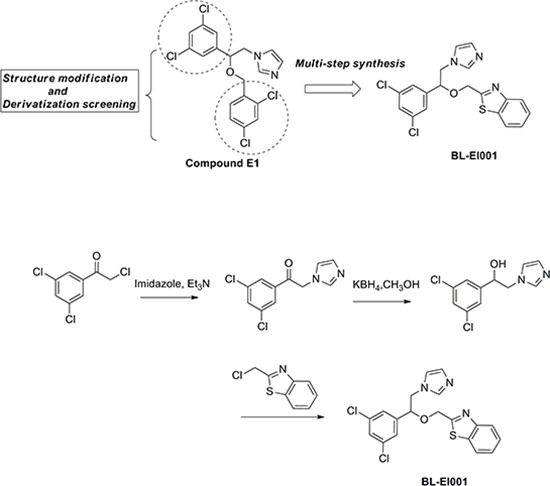
Chemical synthesis of BL-EI001 Chemical structure modification strategies of compound E1, and synthesis of targeted compound BL-EI001 as a novel ERK1/2 inhibitor.

The synthesis of candidate small compounds (compound BL-EI001-BL-EI005): 2-bromo-1-phenylethanone could react with imidazole to produce 2-(1H-imidazol-1-yl)-1-phenylethanoneintermediates in the condition of heating at 70°C and in present of triethylamine, and then the yielding product is condensed with potassium borohydrideby heating at 70°C to give the reduced products, 2-(1H-imidazol-1-yl)-1-phenylethanol intermediates. The condensation of 2-(1H-imidazol-1-yl)-1-phenylethanol intermediates with chloromethyl substituted aromatic compounds in the presence of NaH in refluxing dioxane gives the adduct, which was finally purified by silica-gel column chromatography using PE-EA as an eluent to obtain the final product. The chemical synthesis and structures of BL-EI001-BL-EI005 were shown (Figure 2), and more detailed information was provided as well (Table S3).

**Figure 3:**
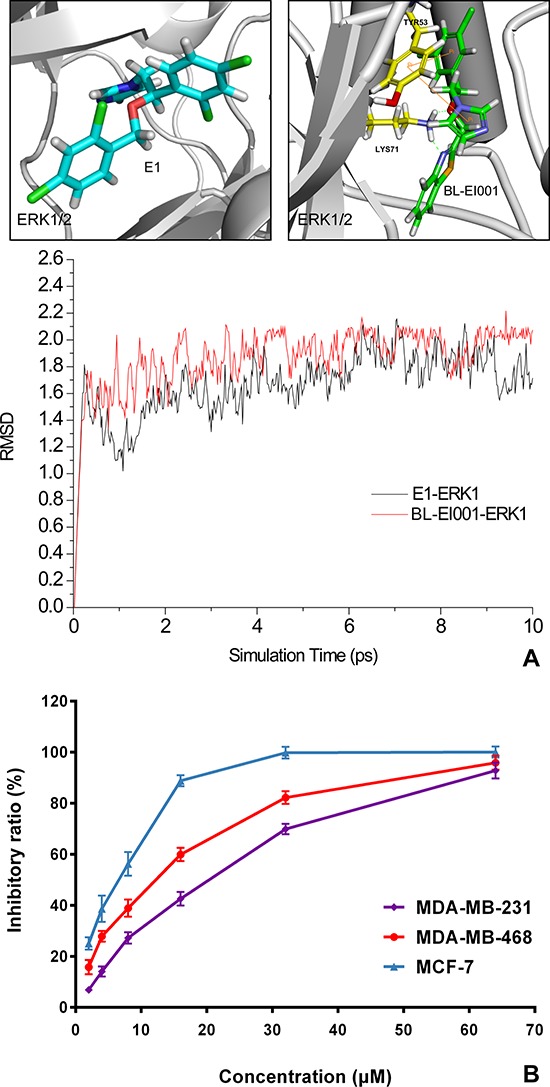
Molecular dynamics (MD) simulation and anti-proliferative activities of E1 and BL-EI001 **(A)** Comparison of molecular dynamics simulation between E1 and BL-EI001 binding to ERK1/2; **(B)** Comparison of anti-proliferative effects between E1 and BL-EI001 in CF-7 cells, MDA-MB468 cells and MDA-MB231 cells, respectively.

From the results of molecular modeling, docking and dynamic stimulation of targeted compounds, we found that the nitrogen of the imidazole ring plays an important role in the interaction with the active sites. Thus, our molecular design retains the pharmacophore imidazole ring (Figure 3A). Docking of new compound BL-EI001 showed that the compound interacted with the active sites of the enzymes through hydrophobic interactions between its aromatic ring and ILE48, VAL56, ALA69 and MET125 amino-acid residues, respectively. Moreover, BL-EI001 formed two hydrogen bonds with the nitrogen of LYS71, and two Pi-Pi interactions with TYR53. (Figure 3A). Therefore, BL-EI001 might have better kinase-binding activity than E1. All the detailed information was shown (Table S4 and Table S5).

### BL-EI001 induces apoptosis in breast adenocarcinoma MCF-7 cells

**Figure 4:**
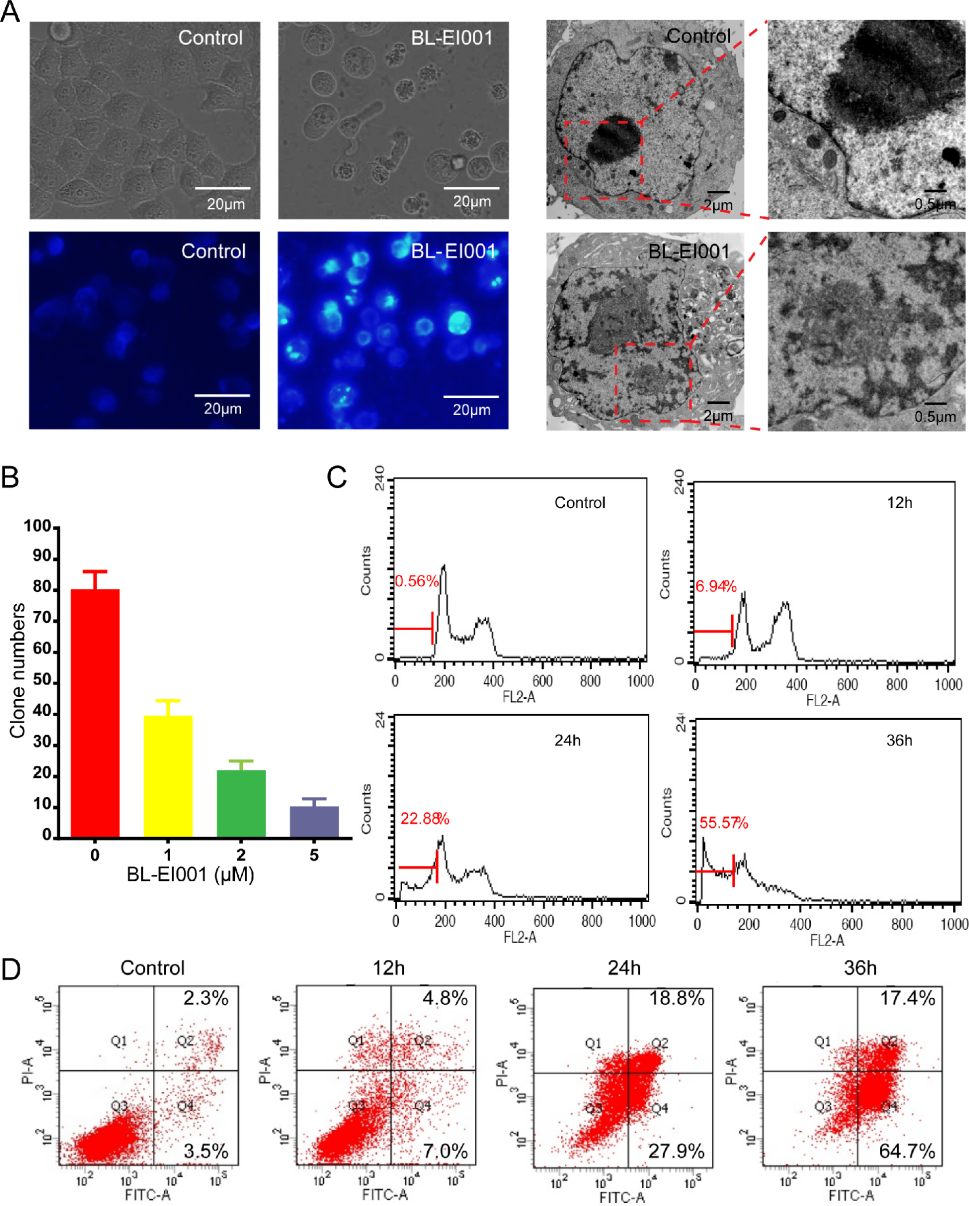
BL-EI001 induces MCF-7 cell apoptosis **(A)** The cellular morphology was observed without or with BL-EI001 under the inverted, fluorescent microscopy and electron microscopy, respectively; **(B)** Cells were consecutively treated with indicated concentrations of BL-EI001 for two weeks. Cell proliferation was examined by colony formation assay; **(C)** The population of SubG1 cells was measured by flow cytometry after collection; **(D)** Apoptosis was detected via Annexin V-FITC/PI staining in MCF-7 cells treated with BL-EI001 for 12, 24, 36 h by flow cytometry analysis.

BL-EI001 caused a significant anti-proliferative effect on MCF-7, MDA-MB468 and MDA-MB231 cell growth in dose-dependent manner, and the treatment with 5 μM BL-EI001 for 24 h resulted in almost 50% inhibition in MCF-7 cells. Also, the treatment with 15 μM and 20 μM BL-EI001 for 24 h resulted in almost 50% inhibition in MDA-MB468 and MDA-MB231 cells, respectively (Figure 3B). To characterize the BL-EI001-induced MCF-7 cell growth inhibition, we observed the morphologic changes in the cells. When the cells were cultured with 5 μM BL-EI001 for 24 h, the apoptotic ultrastructural alterations were also observed under electron microscope. And, the marked apoptotic morphologic alterations were observed by inverted microscopy, as well as by Hoechst 33258 staining under florescence microscopy (Figure 4A). Furthermore, the long-term effects of BL-EI001 on cell survival were determined using a colony formation assay. The results showed that BL-EI001 significantly suppressed cell proliferation, as indicated by the decreased number of cell colonies in the BL-EI001-treated group (Figure 4B). In addition, apoptosis was evaluated by the measurement of cell number in SubG1 region. BL-EI001 markedly induced the increase of SubG1 cells proportion in MCF-7 cells (Figure 4C). Moreover, BL-EI001-induced apoptosis was also measured by flow cytometry after Annexin V-PI staining (Figure 4D). Altogether, these results suggest that BL-EI001 induces apoptosis in MCF-7 cells.

### BL-EI001-induced apoptosis is via the mitochondrial pathway but not dependent on Ras/Raf/MEK1/2 pathway

**Figure 5:**
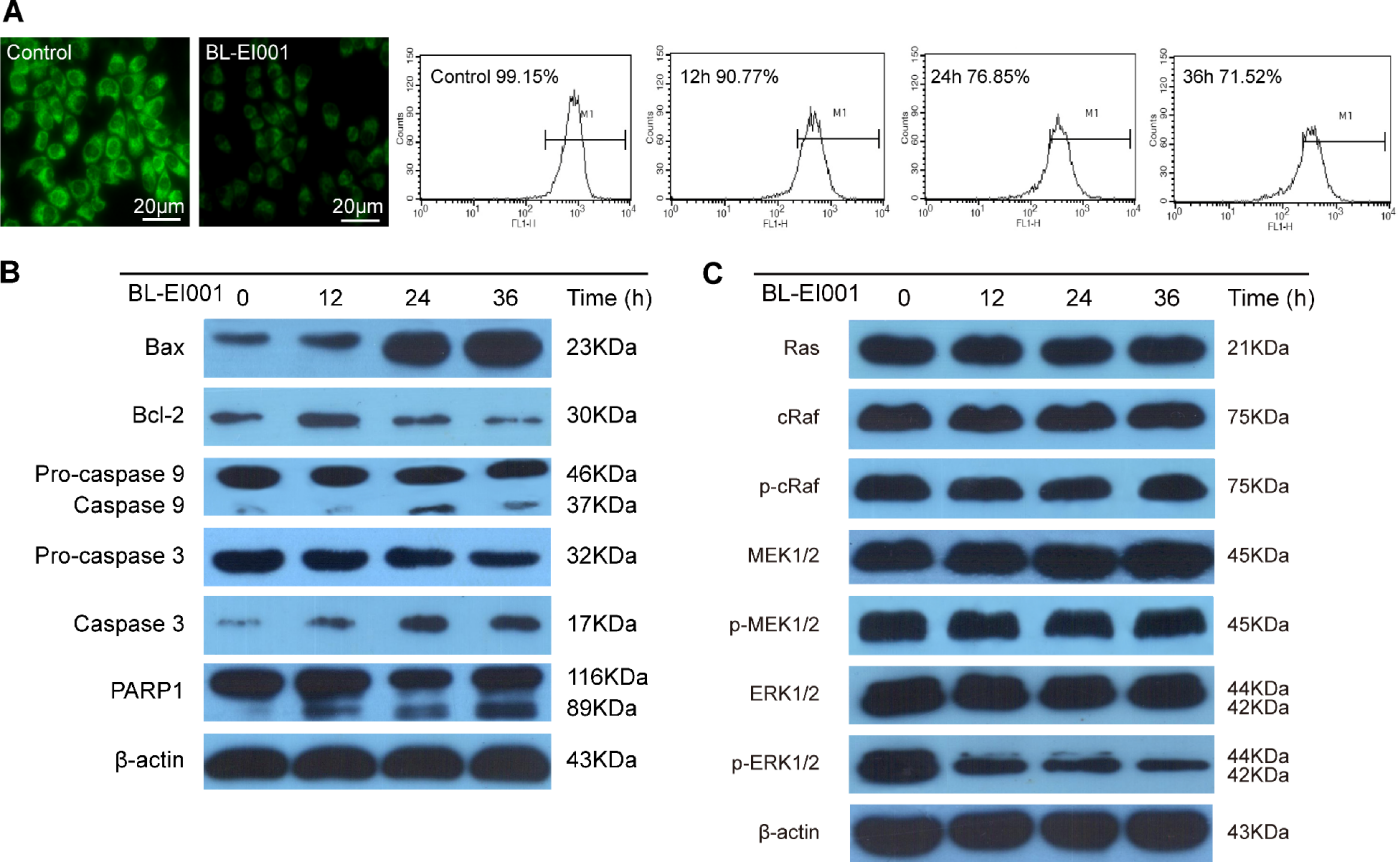
BL-EI001-induced apoptotic mechanism is the mitochondrial pathway but not the Ras-Raf-MEK1/2 pathway **(A)** BL-EI001 treatment resulted in decrease of mitochondrial membrane potential; **(B)** BL-EI001-induced apoptosis activated the mitochondrial pathway; **(C)** BL-EI001-induced apoptosis activated ERK1/2 but not the Ras-Raf-MEK1/2 pathway.

Mitochondrial pathway apoptosis was observed by Rhodamine123 staining and flow cytometry (Figure 5A). Next, Bax expression was increased whereas Bcl-2 expression was decreased in MCF-7 cells (Figure 5B). These results clearly indicate that BL-EI001-induced apoptosis in MCF-7 cells is mediated by the mitochondrial pathway. Then, we investigated the involvements of caspase 9 and caspase 3 in BL-EI001-induced apoptosis. Caspase 9 activation was determined by measurement of the active forms of caspase 9. The active form of caspase 3 was observed during BL-EI001 treatment (Figure 5B). Moreover, we found that the expressions of Ras, c-Raf, p-c-Raf, MEK, p-MEK were not changed in BL-EI001-treated MCF-7 cells, but the expression of p-ERK was remarkably downregulated (Figure 5C). Thus, these results suggest BL-EI001-induced apoptosis is via the mitochondrial pathway but not dependent on Ras/Raf/MEK1/2 pathway.

### Proteomics analyses of BL-EI001-induced apoptotic pathways in MCF-7 and MDA-MB231 cells

**Figure 6:**
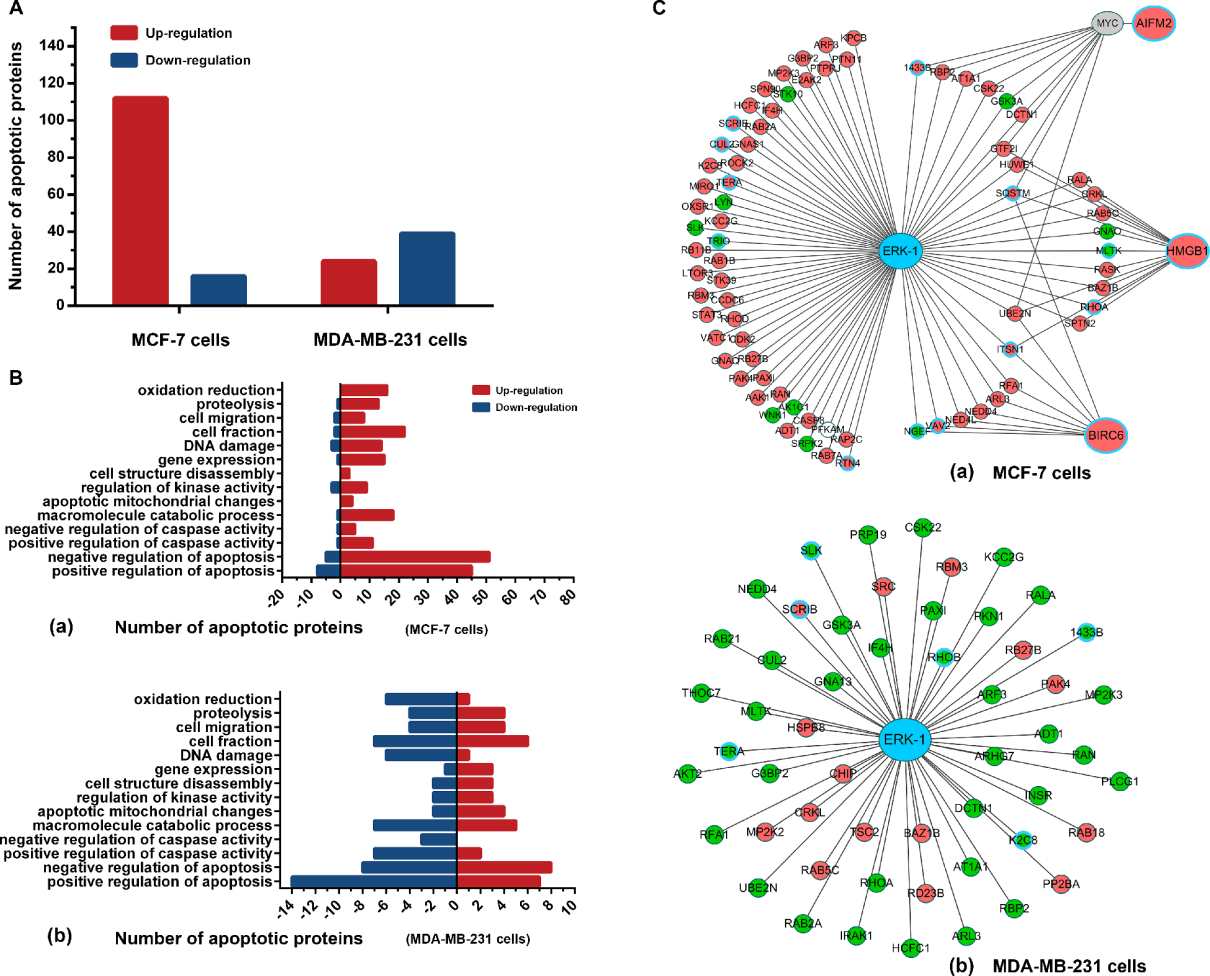
Proteomics analyses of BL-EI001-induced apoptosis in breast cancer cells **(A)** Different protein levels (upregulation or downregulation) between control and BL-EI001-treated MCF-7 and MDA-MB231 cells; **(B)** Apoptosis-related enrichment analyses in BL-EI001-treated MCF-7 and MDA-MB231 cells; **(C)** Proteomics-based identification of novel ERK apoptotic pathways in BL-EI001-treated MCF-7 and MDA-MB231 cells.

To explore the molecular mechanisms underlying BL-EI001-induced apoptosis, iTRAQ and MS/MS analysis was employed to profile differentially expressed proteins in MCF-7 and MDA-MB231 cells treated with BL-EI001, respectively. About thousands of differentially expressed proteins were defined based on both high fold-change and observed in MCF-7 and MDA-MB231 cells (Figure 6A) (Table S6). We annotated them and grouped the 128 apoptosis-related proteins in 14 groups in the two breast cancer cells (Figure 6B). Then, we used these differential expression proteins to construct the ERK network in MCF-7 and MDA-MB231 cells. In MCF-7 cells, three differential expression proteins such as BIRC6, HMGB1 and AIFM2 were predicted to interact with ERK or to be affected by ERK (Figure 6C).

**Figure 7:**
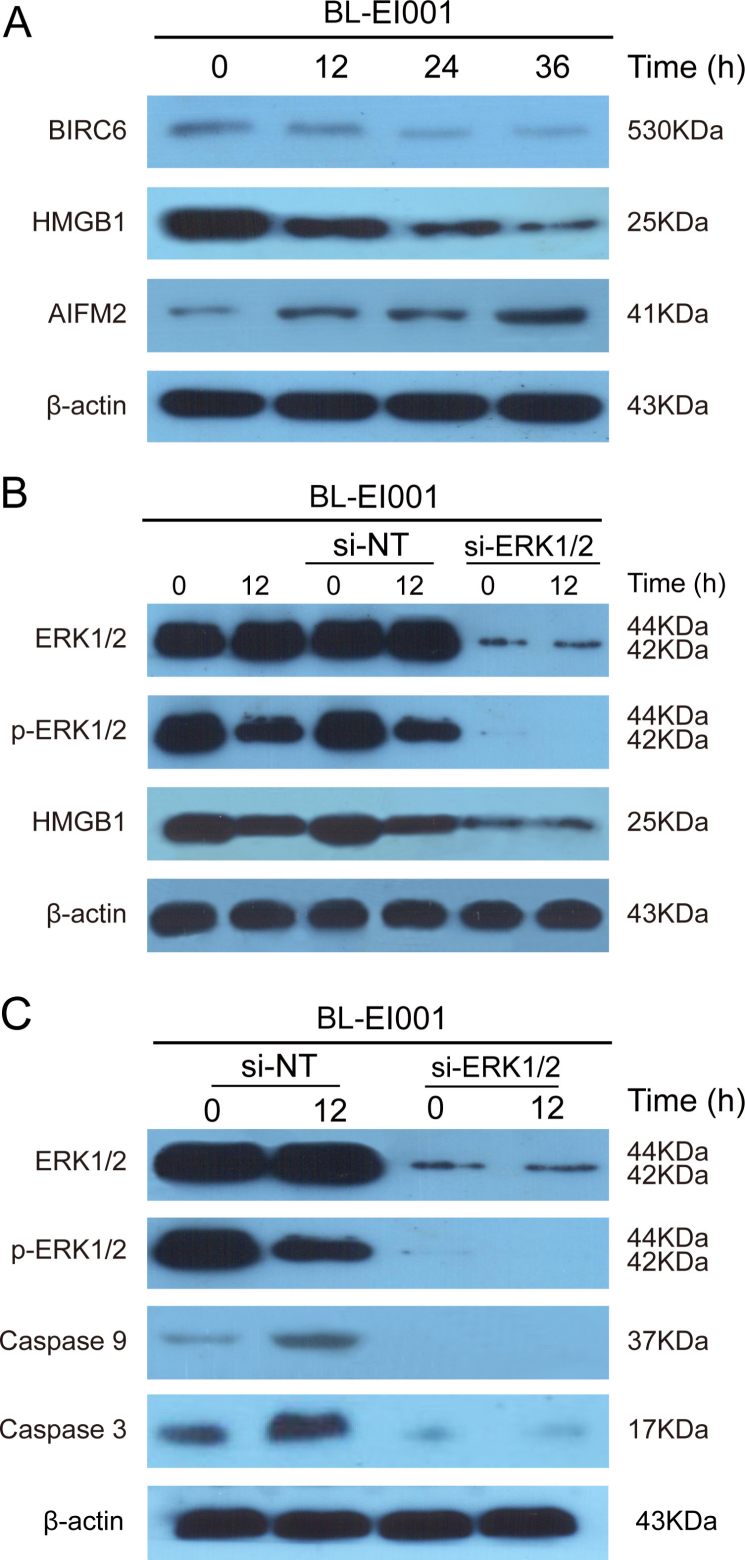
BL-EI001-induced apoptosis is regulated by novel ERK-mediated pathways **(A)** BL-EI001-induced apoptosis is associated with BRIC6, HMGB1 and AIFM2; **(B)** BL-EI001-induced apoptosis is regulated by ERK1/2-HMGB1 pathway; **(C)** BL-EI001-induced apoptosis is affecting ERK1/2.

Next, we found that BIRC6 and HMGB1 were down-regulated whilst AIFM2 was up-regulated after treated with BL-EI001 in MCF-7 cells (Figure 7A). To examine the relationship between ERK and HMGB1, we found that the expression of HMGB1 was decreased significantly while the expression p-ERK1/2 was abruptly decreased under this condition (Figure 7B), suggesting that p-ERK1/2 affect HMGB1 activation in BL-EI001-treated MCF-7 cells. Moreover, we found that p-ERK1/2 expression was significantly decreased in BL-EI001-treated MCF-7 cell apoptosis (Figure 7C). Moreover, caspase 9 and caspase 3 activation are mainly dependent on ERK1/2 phosphorylation in BL-EI001-induced apoptosis and also almost disappeared with siRNA (Figure 7C), suggesting that BL-EI001-induced apoptosis may be dependent on ERK phosphorylation.

### Anti-tumor activity of BL-EI001 *in vivo*

**Figure 8:**
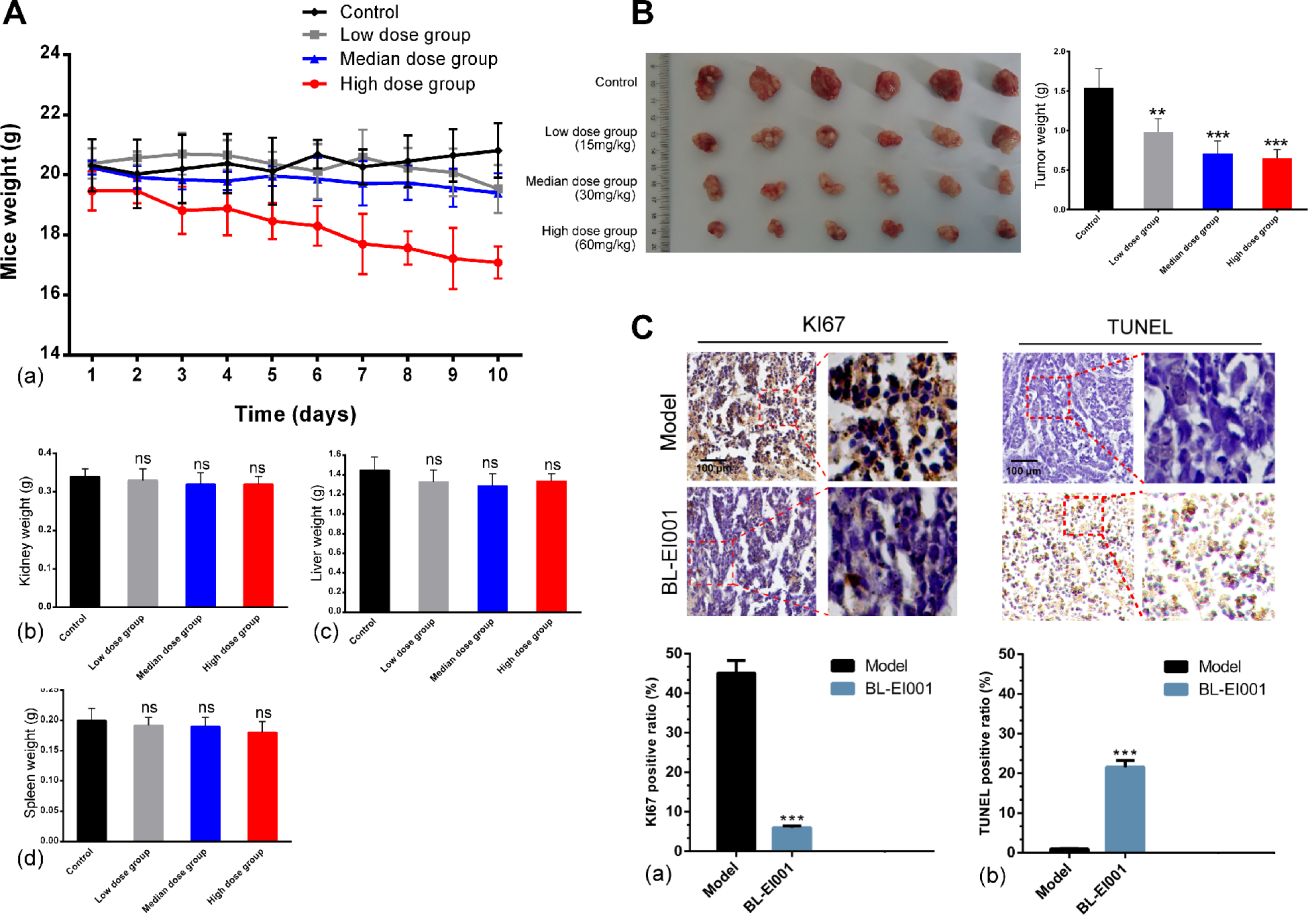
Anti-tumor effects of BL-EI001 *in vivo* **(A)** The toxicity of BL-EI001 in breast cancer xenograft mouse model; **(B)** Illustration shows the tumor excised from each treatment group and data represent the mean tumor weight for each group; **(C)** Immunohistochemistry of proliferative marker KI67 and TUNEL assay.

To evaluate the efficacy of BL-EI001 in MCF-7 cells in nude mice, we used three different doses of BL-EI001. Compared with the control group, median and high BL-EI001 doses can inhibit the growth of human breast tumor significantly in nude mice in a time dependent and dose dependent manner (*P* < 0.001) (Figure 8A). At the end of the experiment, the liver and kidney weights of mice were not affected in all dose groups while the spleen weights were lightly decreased (*P* < 0.01), no other obvious toxicity was observed in mice. We obtained identical results by directly measuring the tumor volumes. In the three BL-EI001 groups, the tumor volumes were much smaller than the control group (Figure 8B). Immunoreactivity for Ki-67, a marker of proliferation, was localized to the cell nuclei. BL-EI001 treatment significantly reduced the number of Ki-67-positive MCF-7 cells compared to the control treatment (Figure 8C), suggesting that BL-EI001-induced apoptosis can inhibit tumor cell proliferation.

**Figure 9:**
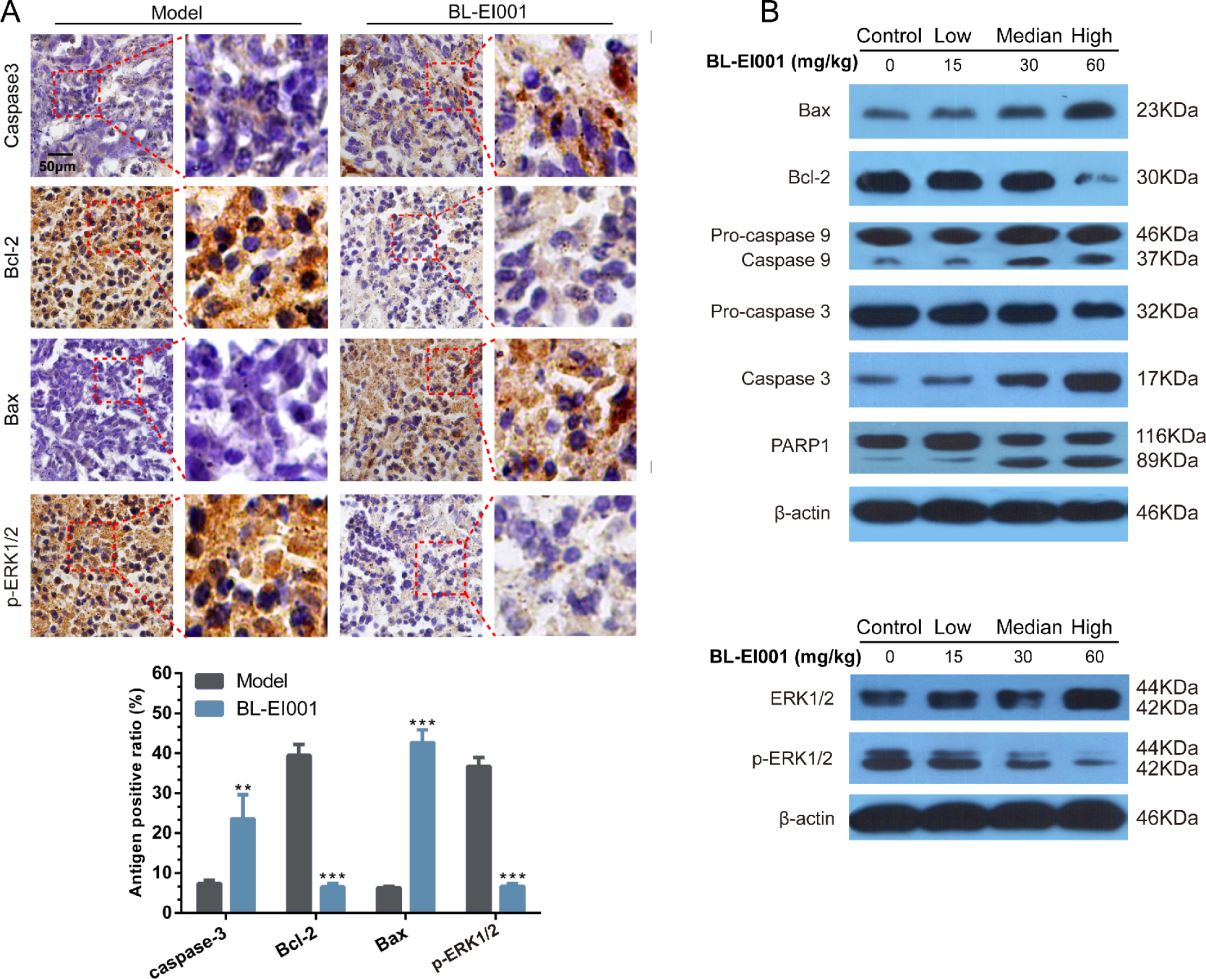
BL-EI001 induces apoptosis *in vivo* **(A)** Immunohistochemistry of p-ERK1/2, Bax, Bcl-2 and cleaved caspase 3 between model- and BL-EI001 groups; **(B)** Some apoptotic marker expressions between control- and different dose-groups; (a) Bax, Bcl-2 and caspase 9/3 and PARP1 expressions; (b) ERK1 and p-ERK1 expressions.

Given the therapeutic efficacy of BL-EI001 in our *in vivo* model, we examined the caspase 3, Bcl-2, Bax and p-ERK1/2 expressions in tumor samples Immunoreactivity. As shown in Figure 6A, active form of caspase 3 was observed in the tumor. And, p-ERK1/2 and Bcl-2 were also inhibited by BL-EI001 whereas Bax expression was increased (Figure 9A). We further demonstrated that both Bax and Bcl-2 expression in BL-EI001-treated tumor samples were consistent with immunohistochemistry results and caspase cascade activation. In addition, DNA repairing protein PARP-1 was sheared significantly (Figure 9B). Moreover, phosphorylation of ERK was inhibited, which was the same as the *in vitro* results. These results demonstrate that BL-EI001 bears remarkable anti-tumor effect, induces apoptosis and affects ERK phosphorylation *in vivo*.

## DISCUSSION

The activity of the Ras-Raf-MEK-ERK cascade has been widely reported to be increased in one-third of human cancers, and inhibition of components of this cascade by targeted inhibitors represents a key therapeutic strategy in cancer [13]. Recent drug discovery has significantly benefited from a rapid progress for understanding how to target protein kinases such as ERK with small-molecule inhibitors in cancer therapy [14]. Previous studies have shown the structure-guided design of potent and selective pyrimidylpyrrole inhibitors of ERK using conformational control and structure–activity relationship studies of 3-(2-amino-ethyl)-5-(4-ethoxybenzylidene)-thiazolidine-2,4-dione as a potential substrate specific ERK1/2 inhibitors [15, 16]. Distinctive from these ERK inhibitors, we computationally screened a series of candidate small-molecule inhibitors from Drugbank and ZINC. Based on the MTT assay of three types of breast cancer cells, we choose a candidate small-molecule compound E1 and synthesized a series of E1 derivatives and eventually found a novel ERK inhibitor (BL-EI001) by further MTT assay in breast cancer cells. Moreover, we used MD simulation to further validate our new synthesized BL-EI001 could bind ERK better than E1, indicating that BL-EI001 is a potent ERK inhibitor. And, we also showed that the anti-proliferative activity of BL-EI001 was greatly improved (IC_50_ = 5 μM, 24 h). Thus, a novel ERK inhibitor (BL-EI001) has been successfully designed and discovered as a candidate anti-tumor drug to possess remarkable anti-proliferative activity toward breast cancer cells by the combination of computational prediction and experimental screening.

Apoptosis is triggered by the extrinsic (death receptor) or intrinsic (mitochondrial) pathway. Caspases are linked to Bcl-2 family which is the key regulator of apoptosis and often over-expresses in cancer [17]. Mitochondrial pathways has been reported that apoptosome-dependent caspase activation depends on the concentration of XIAP, indicating the key roles of procapase 9, Apaf-1, and caspase 3, as well as of a positive feedback loop between caspase 3 and caspase 9 [18, 19]. In our study, we found that the ERK inhibitor (BL-EI001) can induce apoptosis via the mitochondrial pathway in MCF-7 cells. And, we also demonstrated that BL-EI001 induced apoptosis not dependent on Ras/Raf/MEK pathway in MCF-7 cells. Previous study has reported that ERK1/2 docking domain inhibitors can induce apoptosis by targeting Rsk-1 and caspase 9 [20]. And, statins and farnesyl transferase inhibitors have also been reported to affect ERK phosphorylation, and induce apoptosis in non-small lung cancer cells [21]. Moreover, SCH772984, as a selective and potent ERK1/2 inhibitor, has been recently shown to induce apoptosis and cell cycle arrest in BRAF-mutant or non-BRAF-mutant melanoma [11, 12]. To our knowledge, no targeted ERK inhibitor has been reported to be computationally designed, chemically synthesized and experimentally explored its intricate apoptosis-inducing mechanisms in breast cancer. Thus, in our study, we demonstrate that BL-EI001 can induce MCF-7 mitochondrial apoptosis and is independent on Ras/Raf/MEK pathway, suggesting that it may be a potential novel ERK inhibitor in breast cancer.

Moreover, our proteomics experimental data further provides thousands of differential protein expressions in BL-EI001-induced apoptosis toward both MCF-7 and MDA-MB231 cells. Based upon the apoptotic context, novel ERK-regulated apoptotic pathways were identified from thousands of proteins in MCF-7 and MDA-MB231 cells, respectively. These results show that iTRAQ-based identification of potential ERK interactors would be helpful to be utilized for the discovery of more novel apoptotic pathways in breast cancer. In BL-EI001-treated MCF-7 cells, we found ERK might affect BRIC6, HMGB1 and AIFM2. Then, we validated the ERK-HMGB1 pathway was in BL-EI001-treated MCF-7 cells. More importantly, we further found BL-EI001-induced apoptosis was dependent on ERK phosphorylation, suggesting BL-EI001 is a potential targeted ERK inhibitor. Moreover, in our study, we found that EI001 bear the good anti-tumor activities without remarkable toxicities in kidney, liver and spleen, and induced apoptosis by targeting ERK phosphorylation *in vivo*. Interestingly, ERK1/2 inhibition has been reported to enhance apoptosis induced by JAK2 silencing in human gastric cancer SGC7901 cells [22]. In addition, other studies have demonstrated that ERK inhibition overcomes acquired resistance to MEK inhibitors [23, 24]. Thus, combination therapy with some ERK inhibitors and other kinase inhibitors would be utilized as a promising novel strategy for future cancer therapy.

In conclusion, we computationally designed and experimentally identified a novel synthesized ERK inhibitor (BL-EI001) that could induce apoptosis by the mitochondrial pathway but not dependent on Ras/Raf/MEK pathway in breast cancer cells. Based upon further proteomics experiments, we found that EI001-induced apoptosis was associated with some potential ERK interactor involvements, such as HMGB1, BIRC6 and ATFM2 in breast cancer cells. In *in vivo* experiments, we found that EI001 bear the anti-tumor activity without any remarkable toxicity, and also induced apoptosis by the mitochondrial pathway as well as affecting ERK phosphorylation. Therefore, these results may provide a clue for exploiting BL-EI001 as a potential novel small-molecule drug in breast cancer therapeutics.

## MATERIALS AND METHODS

### Docking and molecular dynamics (MD) stimulations

The initial three dimensional geometric coordinates of the X-ray crystal structure of ERK1/2 (4QTB) was downloaded from the Protein Data Bank (PDB) (http://www.pdb.org/pdb/home/home.do). And, we constructed the screening library for ERK1/2 containing all FDA-approved small molecule compounds from the latest version of Drugbank (http://www.drugbank.ca/) and ZINC (http://www.zinc.docking.org/), respectively. Additionally, we used DOCK6 to dock the pre-generated conformations of drugs into ERK1/2 for virtually screening their inhibitors [25]. We performed flexible-ligand docking to a rigid receptor with grid-based scoring, in which the ligands (drugs) were allowed to be flexible and structurally rearranged in response to the receptor (ERK1/2). Subsequently, we selected 11 small molecules to validate their anti-proliferative activities. Moreover, MD stimulations were performed with GROMACS (version 4.5.5) software package to monitor the binding states between ERK1/2 and the selected BL-EI001 [26].

### Chemical synthesis of candidate compounds targeting ERK

All reagents were purchased from commercial sources and used without further purification. Melting points are corrected. ^1^H-NMR spectra were determined on a Bruker Avance III 400MHz spectrometer in CDCl^3^ or DMSO-d^6^ solution. J values are in Hz. Chemical shifts are expressed in ppm downfield from internal standard TMS. HRMS data were obtained using Bruker micro-TOF-Q instrument or TOF-MS instrument. The synthesis of candidate small compounds (compound BL-EI001-BL-EI005): 2-chloro-1-(2,4-dichloro-phenyl) ethanone could react with imidazole to produce 2-(1H-imidazol-1-yl)-1-phenylethanone intermediates in the condition of heating at 70°C and in present of triethylamine, and then the yielding product is condensed with potassium borohydride by heating at 70°C to give the reduced products, 2-(1H-imidazol-1-yl)-1-phenylethanol intermediates. The condensation of 2-(1H-imidazol-1-yl)-1-phenylethanol intermediates with chloromethyl substituted aromatic compounds in the presence of NaOH and PEG600 in refluxing dioxane gives the adduct, which was finally purified by silica-gel column chromatography using PE-EA as an eluent to obtain the final product.

### Cell culture and reagents

Human breast adenocarcinoma MCF-7, MDA-MB468 and MDA-MB231 cells were purchased from American Type Culture Collection (ATCC, Manassas, VA, USA). They were routinely cultured in DMEM containing 10% fetal bovine serum, 100 U/ml streptomycin, 100 U/ml penicillin, and 2 mM L-glutamine in a humidified cell incubator with an atmosphere of 5% CO_2_ at 37°C. Antibodies against Bax, Bcl-2, caspase-3, caspase-9, PARP1, Ras, c-Raf, p-cRaf, MEK1/2, p-MEK1/2, ERK1/2, p-ERK1/2, BIRC6, HMGB1, AIFM2, Ki-67 and β-actin, HRP-conjugated secondary antibodies and siRNA against human ERK1/2 and control siRNA were purchased from Cell Signaling Technology. Hoechst 33258 and Rhodamine 123 were purchased from Sigma. *In Situ* Cell Death Detection Kit, POD (TUNEL) and Annexin-V-FLUOS Staining Kit were purchased from Roche.

### Cell viability assay

Human breast adenocarcinoma MCF-7, MDA-MB468 and MDA-MB231 cells were dispensed in 96-well flat bottom microtiter plates at a density of 5 × 10^4^ cells/ml. After 24 h incubation, they were treated with different concentrations of E1-E11 and BL-EI001 for the indicated time periods, respectively. Cell viability was measured by the 3-(4, 5-dimetrylthiazol-2-yl)-2, 5-diphenyltetrazolium bromide (MTT) assay.

### Apoptosis assay

The MCF-7 cells were seeded into 6-well culture plates with or without BL-EI001 and cultured for 24 h. The ultrastructure of cell apoptosis was observed under the electron microscope (Hitachi7000, Japan). For colony formation assay, 150 counted cells were seeded in a 60 mm dish. The cells were continuously cultured in presence or absence of indicated concentration of BL-EI001 for 14 days. Clones were stained with Giemsa and counted by using a microscope. Only those cell clusters containing more than 50 cells was considered as a clone. The collected cells were fixed with 500 μL PBS and 10 ml 70% ethanol at 4°C overnight; then after washing twice with PBS, the cells were incubated with 1 ml Hoechst 33258 or Rhodamine123 staining solution for 30 min at 4°C. For Annexin V-FITC/PI staining, the treated cells were collected, washed and then stained with Annexin V-FITC/PI at room temperature for 15 min. The percentage of apoptotic cells and cells at different phases of the cell cycle or the Sub-G1 DNA content was measured by flow cytometry (Becton Dickinson, Franklin Lakes, NJ).

### Western blot analysis

The MCF-7 cells were treated with 5 μM/ml BL-EI001 for 0, 12, 24 and 36 h, respectively. Both adherent and floating cells were collected, and then western blot analysis was carried out. Briefly, the cell pellets were resuspended with lysis buffer consisting of Hepes 50 mmol/L pH 7.4, Triton-X-100 1%, sodium orthovanada 2 mmol/L, sodium fluoride 100 mmol/L, edetic acid 1 mmol/L, PMSF 1 mmol/L, aprotinin (Sigma, MO, USA) 10 mg/L and leupeptin (Sigma) 10 mg/L and lysed at 4°C for 1 h. After 12,000 × g centrifugation for 15 min, the protein content of supernatant was determined by the Bio-Rad DC protein assay (Bio-Rad Laboratories, Hercules, CA, USA). Equal amounts of the total protein were separated by 10–15% SDS-PAGE and transferred to nitrocellulose membranes, the membranes were soaked in blocking buffer (5% skimmed milk). Proteins were detected using primary antibodies, followed by HRP-conjugated secondary antibody and visualized by using ECL as the HRP substrate.

### SiRNA transfection

Small interfering RNAs (siRNAs) against human ERK1/2 was purchased from Cell Signaling Technology. The MCF-7 cells were transfected with siRNAs at 100 nM final concentration using Lipofectamine 2000 (Invitrogen) according to the manufacturer's instructions. The transfected cells were used for subsequent experiments 24 h later.

### iTRAQ and MS/MS analysis

Two separate iTRAQ and MS/MS analysis were performed in BL-EI001-treated MCF-7 and MDA-MB231 cells. Briefly, cells were dissolved in lysis buffer in presence of protease inhibitor (Sigma). The lysate was centrifuged for 1 h at 15°C and the supernatant was stored at −80°C for further use. Protein quantitation was performed using RCDC Protein Assay Kit (Bio-Rad). iTRAQ labeling was carried out using iTRAQ Reagent 4-Plex kit (AB SCIEX) based on the manufacturer's protocol with minor modifications. A pair of HCT116-Control and HCT116-CacyBP OE whole cell lysates were labeled with iTRAQ labeling reagent 113 and 114 for the pair of control MCF-7 and BL-EI001-treated MCF-7 whole cell lysate, respectively. Control MDA-MB-231 and BL-EI001-treated MDA-MB-231 cells were labeled with 114 and 115 iTRAQ labeling reagents, respectively. After 2D LC analysis and tandem mass spectrometry analysis, protein identification and relative iTRAQ quantification were performed with ProteinPilot™ Software 4.2 (AB SCIEX) using the Paragon™ algorithm for the peptide identification, which was further processed by Pro GroupTM algorithm where isoform-specific quantification was adopted to trace the differences between expressions of various isoforms. Results with iTRAQ ratio cutoff values of 1.2 and 0.8 for fold-change and number cutoff values of 3 for quantifiable peptides for in protein abundance were accepted.

### Functional enrichment analyses

Differentially expressed proteins were assigned to 14 functional synaptic protein groups. Enrichment was only considered relevant when over represented functional groups contained at least 5 proteins. In addition, functional enrichment was determined using the DAVID functional annotation tool (http://david.abcc.ncifcrf.gov/). The functional categories used were GO term related to Biological Process (BP), Cellular Component (CC), and Molecular Function (MF), as well as pathway annotations derived from KEGG. And, the apoptosis-related proteins was detected and used as the background set.

### Mouse experiments and tumor xenograft model

Healthy female nude mice (BALB/c, 6–8 weeks of age, non-fertile, 18–20 g of weight) were injected subcutaneously with MCF-7 cells (1 × 10^7^ cells/mouse). When the tumors reached 100 mm^3^ in volume (calculated as V = L × W^2^/2), mice were treated with either hydroxypropyl-cyclodextrin (vehicle control) or BL-EI001 (low dose group: 15 mg/kg oral twice daily; median dose group: 30 mg/kg oral twice daily; high dose group: 60 mg/kg oral twice daily). Animals were sacrificed after 10 days. Tumor tissues, were isolated and frozen in liquid nitrogen or fixed in formalin immediately.

### Immunohistochemistry and TUNEL assay

Immunohistochemistry was performed. Briefly, sections were dewaxed and rehydrated. Antigen retrieval was performed by pretreatment of the slides in citrate buffer (pH 6.0) in a microwave oven for 12 min. The slides were incubated with PBS containing 3% hydrogen peroxide for 15 min, PBS containing 5% BSA for 30 min, and subsequently the primary antibody at 4°C overnight. The slides were then probed with horseradish peroxidase-conjugated secondary antibody for 1 h at 37°C, followed by reaction with diaminobenzidine and counterstaining with Mayer's hematoxylin. To score each slide, at least eight individual fields were chosen and 100 cells were counted in each field. Cells with Ki-67, caspase3, Bcl-2, Bax and p-ERK1/2 immunoreactivity were considered positive. For TUNEL assay, sections were permeabilized with 0.1% Trition X-100 plus 0.1% sodium citrate and then incubated with 50 μL TUNEL reaction mixture (Roche) at 37°C for 60 min. After rinsing with PBS three times, 50 μL converter-POD was added and the tissue cells were incubated in a humidified chamber for 30 min at 37°C. DAB substrate was then added, followed by counterstaining with hematoxylin. The assay included negative controls (without terminal transferase). Apoptosis was quantified by counting the number of TUNEL-positive cells in at least six non-overlapping high-power fields on each section and evaluated.

### Statistical analysis

All the presented data and results were confirmed in at least three independent experiments. The data are expressed as means ± S.D. Statistical comparisons were made by Student's *t*-test. *P* < 0.05 was considered statistically significant.

## SUPPLEMENTARY TABLES




