# Supplementary material for: Computational design, chemical synthesis, and biological evaluation of a novel ERK inhibitor (BL-EI001) with apoptosis-inducing mechanisms in breast cancer
Source: Oncotarget. 2015 Jan 27;6(9):6762–75. doi: 10.18632/oncotarget.3105 (PMC4466648; doi:10.18632/oncotarget.3105)
Supplement: Supplementary file 1 [file oncotarget-06-6762-s001.pdf]

SUPPLEMENTARY TABLES

Supplementary Table S1: Screening candidate small-molecule compounds from Drugbank

Rank	Zinc	Energy	Popular name
1	C00599734	–45.22	Lansoprazole
2	C03831448	–40.49	Silibinin (INN)
3	C03830847	–39.64	Flubendazole
4	C03830270	–39.62	methyl
5	C01757652	–39.31	Silibinin (INN)
6	C01713761	–38.94	Nialamide
7	C00607971	–38.85	Miconazole Nitrate
8	C00897408	–38.79	Bicalutamide
9	C01530580	–38.13	Carvedilol
10	C00020260	–38.05	Oxyphencyclimine Hydrochloride
11	C03830179	–37.77	9-pentofuranosyl-9H-purin-6-amine
12	C03831401	–37.36	Pyrvinium pamoatemethyl
13	C03831198	–37.31	methyl
14	C00000061	–37.11	Baclofen
15	C01039227	–37.04	
16	C00057439	–36.58	Oxyphencyclimine Hydrochloride
17	C04676424	–36.55	Pantoprazole
18	C00057439	–36.28	Oxyphencyclimine Hydrochloride
19	C03830179	–36.27	9-pentofuranosyl-9H-purin-6-amine
20	C00005895	–36.07	Rizatriptan Benzoate

Supplementary Table S2: Screening candidate small-molecule compounds from ZINC

Rank	Zinc	Energy	Popular name
1	C00599734	–45.22	2-[[3-methyl-4-(2,2,2-trifluoroethoxy)-2-pyri]]
2	C03831448	–40.49	3,5,7-trihydroxy-2-[8-(4-hydroxy-3-methoxy-ph
3	C03830847	–39.64	methyl
4	C03830270	–39.62	methyl
5	C01757652	–39.31	3,5,7-trihydroxy-2-[8-(4-hydroxy-3-methoxy-ph
6	C01713761	–38.94	N-benzyl-3-(N'-(4-pyridylcarbonyl)hydrazino)-
7	C00607971	–38.85	1-[2-(2,4-dichlorophenyl)-2-[(2,4-dichlorophe
8	C00897408	–38.79	N-[4-cyano-3-(trifluoromethyl)phenyl]-3-(4-fl
9	C01530580	–38.13	1-(9H-carbazol-4-yloxy)-3-[2-(2-methoxyphenox
10	C00020260	–38.05	(1-methyl-5,6-dihydro-4H-pyrimidin-2-yl)methy
11	C03830179	–37.77	2-(6-aminopurin-9-yl)-5-(hydroxymethyl)tetrah
12	C03831401	–37.36	2-[2-(2,5-dimethyl-1-phenyl-pyrrol-3-yl)vinyl
13	C03831198	–37.31	methyl
14	C00000061	–37.11	4-amino-3-(4-chlorophenyl)-butanoic
15	C01039227	–37.04	5-methoxy-2-[(4-methoxy-3,5-dimethyl-2-pyridy
16	C00057439	–36.58	(1-methyl-5,6-dihydro-4H-pyrimidin-2-yl)methy
17	C04676424	–36.55	5-(difluoromethoxy)-2-[(3,4-dimethoxy-2-pyrid
18	C03830179	–36.27	2-(6-aminopurin-9-yl)-5-(hydroxymethyl)tetrah
19	C00005895	–36.07	N,N-dimethyl-2-[5-(1,2,4-triazol-1-ylmethyl)-
20	C00000197	–35.95	N,N-bis(2-chloroethyl)-2-oxo-1-oxa-3-aza-2$l^

Supplementary Table S3: Modified candidate small-molecule compounds from BL-EI001 to BL-EI005

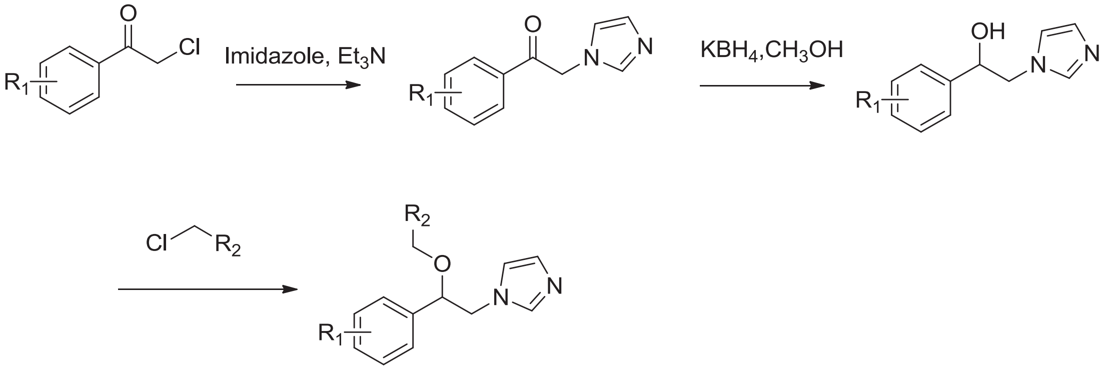

BL-EI001~ BL-EI005

Compound	Structure	Characterization data
BL-EI001	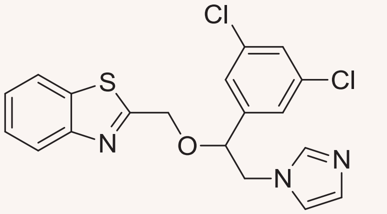	^{1}H NMR (400 MHz, $CDCl_3$) δ 8.18 (d, *J* = 17.9 Hz, 1H), 8.02 (d, *J* = 18.1 Hz, 1H), 7.92 (s, 1H), 7.59 – 7.48 (m, 3H), 7.34 (d, *J* = 3.0 Hz, 2H), 7.18 (d, *J* = 15.0 Hz, 1H), 6.81 – 6.74 (m, 1H), 5.65 (s, 2H), 5.36 (t, *J* = 8.9 Hz, 1H), 4.77 (dd, *J* = 24.7, 8.8 Hz, 1H), 4.24 (dd, *J* = 24.7, 8.8 Hz, 1H). ^{13}C NMR (100 MHz, $CDCl_3$) δ 166.48, 152.11, 143.89, 139.84, 135.76, 128.74, 126.89, 126.28, 124.60, 123.49, 121.34, 120.63, 80.76, 63.31, 50.35. HRMS(m/z) = 404.0399 (M + H)
BL-EI002	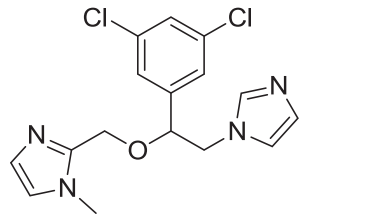	^{1}H NMR (400 MHz, $CDCl_3$) δ 7.90 (s, 1H), 7.54 (s, 1H), 7.33 (d, *J* = 2.9 Hz, 2H), 7.17 (d, *J* = 15.2 Hz, 1H), 6.87 – 6.73 (m, 3H), 5.42 (s, 1H), 4.76 (s, 2H), 3.72 (s, 3H). ^{13}C NMR (125 MHz, 100 MHz, $CDCl_3$) δ 143.89, 143.25, 139.84, 135.68, 128.74, 127.19, 126.89, 124.60, 120.63, 120.33, 80.76, 64.61, 50.35, 31.96. HRMS(m/z) = 373.0604 (M + Na)
BL-EI003	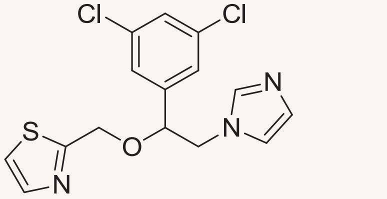	^{1}H NMR (400 MHz, $CDCl_3$) δ 7.92 (s, 2H), 7.71 (d, *J* = 15.0 Hz, 2H), 7.56 (s, 2H), 7.34 (dd, *J* = 8.9, 5.9 Hz, 6H), 7.18 (d, *J* = 15.2 Hz, 2H), 6.78 (d, *J* = 14.6 Hz, 2H), 5.60 (s, 1H), 4.91 (s, 4H). ^{13}C NMR (100 MHz, $CDCl_3$) δ 165.36, 143.89, 139.84, 139.57, 135.68, 128.74, 126.89, 124.60, 120.63, 117.16, 80.76, 62.27, 50.35. HRMS(m/z) = 345.0241 (M + H)
BL-EI004	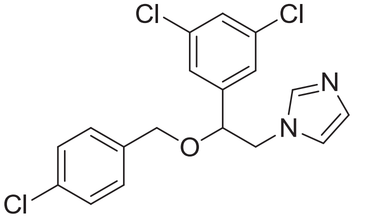	^{1}H NMR (400 MHz, $CDCl_3$) δ 7.92 (s, 1H), 7.56 (t, *J* = 1.4 Hz, 1H), 7.36 (dd, *J* = 15.4, 4.5 Hz, 4H), 7.28 (d, *J* = 7.5 Hz, 2H), 7.18 (d, *J* = 7.4 Hz, 1H), 6.78 (d, *J* = 7.5 Hz, 1H), 5.20 (t, *J* = 4.9 Hz, 1H), 4.64 (s, 2H), 4.24 (d, *J* = 4.8 Hz, 1H), 4.17 (d, *J* = 4.8 Hz, 1H). ^{13}C NMR (100 MHz, $CDCl_3$) δ 143.89, 139.84, 137.55, 135.68, 135.14, 129.75, 128.74, 128.29, 126.89, 124.60, 120.63, 80.49, 71.81, 50.35. HRMS(m/z) = 381.0338 (M + H)
BL-EI005	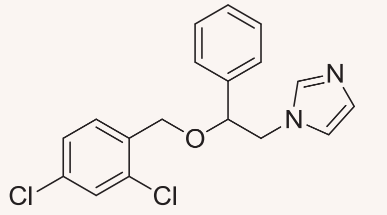	^{1}H NMR (400 MHz, $CDCl_3$) δ 7.92 (s, 1H), 7.75 (d, *J* = 2.5 Hz, 1H), 7.41 – 7.10 (m, 8H), 6.87 – 6.69 (m, 1H), 5.16 (t, *J* = 9.5 Hz, 1H), 4.76 (s, 2H), 4.32 (dd, *J* = 24.7, 9.5 Hz, 1H), 4.14 (dd, *J* = 24.9, 9.5 Hz, 1H). ^{13}C NMR (100 MHz, $CDCl_3$) δ 141.22, 139.84, 135.85, 135.64, 134.25, 130.82, 129.77, 128.62, 127.17 (s), 126.84, 120.63, 80.98, 67.64, 50.35. HRMS(m/z) = 369.0540 (M + Na)

Supplementary Table S4: Binding free energies and individual energy terms of E1 and BL-EI001 in complex with ERK (kcal/mol)

Contribution	E1	BL-EI001
ΔE_{int}^{ele}	–15.30(0.49)	–3.15(0.17)
ΔE_{int}^{vdw}	–34.56(0.37)	–43.35(0.33)
ΔG_{sol}^{nopol}	–4.71(0.02)	–5.37(0.05)
ΔG_{sol}^{ele}	25.63(0.38)	16.44(0.23)
ΔG_{sol} [a]	20.92(0.36)	11.07(0.21)
ΔG_{ele} [b]	10.33(0.45)	13.29(0.38)
–TΔS	–8.5(0.12)	–9.6(0.18)
ΔG_{bind}	–37.15(0.42)	–45.02(0.36)

[a]The polar/nonpolar ($\Delta G_{sol}^{ele}+\Delta G_{sol}^{nopol}$) contributions.
[b]The electrostatic ($\Delta E_{int}^{ele}+\Delta G_{sol}^{ele}$) contributions. All energies are averaged over 200 snapshots and are given in kcal/mol. Calculation of ΔG_{bind} does not explicitly consider entropy contributions. The values in parentheses represent the standard error of the mean.

Supplementary Table S5: Hydrogen bonds analysis of the BL-EI001 inhibitors into ERK binding site based upon MD simulation

Complex	Donor	Acceptor-H	Acceptor	% Occupied	Distance(Å)	Angle(Degree)
BL-EI001-ERK	352:N5	48:HZ3	48:NZ	23.0	2.880	27.19
	352:N5	48@HZ2	48:NZ	23.0	2.878	28.96
	352:N5	48@HZ1	48:NZ	14.6	2.869	26.93

Supplementary Table S6: Proteomics-based identification of differential protein expressions in BL-EI001-induced apoptosis in MCF-7 and MDA-MB231 cells
